# Tracking domestication signals across populations of North American raccoons (*Procyon lotor*) via citizen science-driven image repositories

**DOI:** 10.1186/s12983-025-00583-1

**Published:** 2025-10-02

**Authors:** Artem Apostolov, Alanis Bradley, Shane Dreher, Cole Dwyer, Jessica Edwards, Marie E. Evans, Nari Gu, Jacob Hansen, Jackson D. Lewis, Aiden T. Mashburn, Kelsey Miller, Eli Richardson, Wesley Roller, Adam Stark, Jackson Swift, Oscar Zuniga, Raffaela Lesch

**Affiliations:** Department of Biology, University of Arkansas at Little Rock, Little Rock, USA

**Keywords:** Domestication, *Procyon lotor*, Neural crest, Commensalism, Tameness, Urbanization, NCDS

## Abstract

North American raccoons are widespread across the contiguous United States and live in close proximity to humans (i.e. urban) and in rural environments. This makes them an excellent species for comparative work on the effects of human environments on phenotypic traits. We use raccoons as a mammalian model system to test whether exposure to human environments triggers a trait of the domestication syndrome. Our data suggests that urban environments produce reductions in snout length, which are consistent with the domestication syndrome phenotype. These results are crucial for the discussion of the validity of the Neural Crest Domestication Syndrome hypothesis. They also offer new opportunities to potentially observe early-stage domestication patterns in a yet non-domesticated mammalian species, without the possibility of introgression or hybridization with other already domesticated mammals.

## Background

Domestication is often misunderstood as a purely human-driven “unnatural” process of artificial selection, a view that could not be more inaccurate [8]. The process of domestication across species starts with the adaptation of a subpopulation to a new environmental niche in the human environment [2, 11]. The combination of the ready availability of refuse, i.e., food scraps, and the absence of other large predators make the human environment a niche of great potential [47]. To best exploit this specific environment, animals would have to adapt to interference from humans: caution and care were necessary, but more importantly, only animals with dampened flight (or fight) responses would succeed best [35]. This makes the initial stages of the domestication process a process of pure natural selection.

**Fig. 1:**
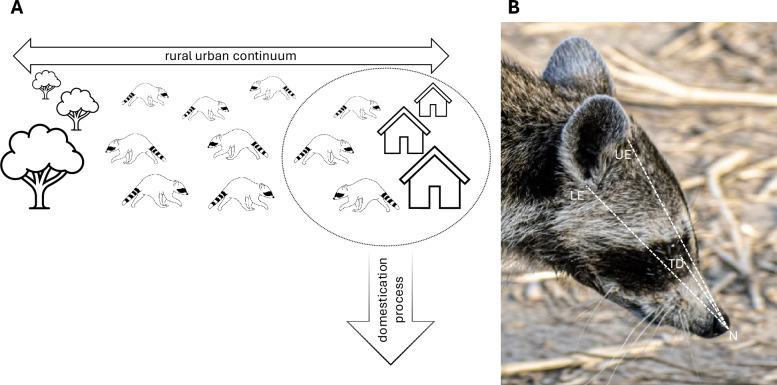
**A** Illustration of the concept of comparing urban and rural raccoon populations. Raccoons living in urban environments should experience selection pressures found in the beginning stages of the domestication process. **B** Snout and skull length measurements measured on all the images. Snout length is measured from the most rostral tip of the nose (N) to the tear duct (TD) of the eye. The skull length proxy is measured by averaging the length of the distance between the rostral tip of the nose (N) to the base of the lower (LE) and upper pinna attachment (UE), resulting in a proxy of skull length centering at the ear canal opening

Only more recently did domesticated animals start to be subjected to selective, human-driven breeding that initially resulted in land races (i.e., animals with a specific purpose yet diverse looks), which eventually turned into what we now know as well-established pedigree breeding programs focused on morphological traits [28]. Crucially, any comparison between domesticated representatives of a species and their wild cousins (think wolves and dogs) will result in pitfalls in the comparison of two groups that have experienced severe artificial selection pressures on one side (e.g., breeds such as pugs and German shepherds) and continuous natural selection pressures as well as introgression and hybridization via domestic individuals on the other side. To disentangle the (early) domestication signal from any other signals, such as genetic drift, bottle necking, or inbreeding, we need to compare subpopulations in the initial stages of domestication (i.e., proto domesticates, Fig. 1A) to subpopulations not impacted by the same selection pressures [22, 29, 37]. This study aims to investigate the initial impact of domestication on mammalian skull anatomy.

Current domesticated animals share patterns and traits that are ubiquitous across the phylogenetic tree; these traits and similarities are summarized under the umbrella term “domestication syndrome” [18, 46]. Anatomical and morphological changes such as curly tails, floppy ears, depigmentation, smaller brains and reduced facial skeletons are commonly cited as some of the most salient traits. These ubiquitous patterns of change have inspired the Neural Crest Domestication Syndrome (NCDS) hypothesis, which suggests a mechanistic pathway tied to the neural crest in early embryonic development [45, 46]. It outlines that (passive) selection for tameness in the process of domestication, i.e., adaptation to a human environment, has altered and reduced the number of neural crest cells arriving at target sites [7]. This reduction in progenitor cells has the potential to explain all “reductions” we commonly observe in the domestication syndrome, such as a lack of pigmentation (i.e., white patches), smaller brains, smaller teeth, and floppy ears [5, 15, 27].

North American raccoons (*Procyon lotor*) are widely distributed across the continental United States and are readily found both in rural and urban environments [16, 33]. Populations in urban environments live in close contact with humans and make excellent use of this specific niche: racoons are omnivorous and feed on plant material, insects, crustaceans, fish, and small mammals but also, crucially, garbage [17]. In densely populated urban environments there is much opportunity to exploit human refuse-related food sources. According to our scientific understanding of domestication events, these populations experience intense selection pressures for reduced reactivity towards humans in order to best exploit their environment [34, 44]. This pattern of adaptation to the human environment mimics the environment in which most commensal domesticates, such as dogs and cats would have found themselves in [47].

In this manuscript, we test whether raccoon populations in close proximity to humans exhibit phenotypic traits of the domestication syndrome. More specifically, we predict that urban raccoons have shorter snouts than rural populations. To test this prediction, we use image data (N = 19,495) of raccoons collected through iNaturalist across the entire continental United States.

## Methods

### Image source and selection

All raccoon images were sourced via iNaturalist (https://www.inaturalist.org/), a free website/application based on the concept of citizens uploading images of fauna and flora. The community then helps identify the species of the uploaded image, and the picture is made available for research via the Global Biodiversity Information Facility (https://www.gbif.org/). The data obtained through this process are used through the Creative Commons Public Licenses (http://creativecommons.org/licenses/by-nc/4.0/legalcode). The complete dataset used in this study is made available through the Global Biodiversity Information Facility [21],doi in reference). Images taken in the United States ranging from 2000 to 2024 (including up to August 12th) were pulled from the repository, resulting in a total of 105,722 images.

### Image criteria

Individual members of the platform can contribute unlimited amounts of pictures to the repository. To control for potential duplicates of individual raccoons (i.e., multiple pictures uploaded by the same person photographing the same raccoon), we reduced the image contribution to one individual picture per person. This reduced the dataset from 105,722 to 19,495 pictures, which is a reduction to _˜_18.4% of the original dataset. The smaller dataset of 19,495 images was then randomly divided into 17 datasets of approximately equal size; each dataset was assigned to an author who was blind to the conditions (e.g. rural vs urban, location) of each image. Each of the 17 authors would then manually go through their assigned dataset of approximately 1,140 images to decide whether a specific image met all of the following criteria: (I) a living (or very recently deceased) raccoon is visible, (II) the raccoons head is oriented in profile view, (III) the head is visible in its entirety, (IV) the image resolution/quality is high enough to allow visual identification of anatomical landmarks, and (V) the individual present is a member of the correct species (i.e., *Procyon lotor*). After successful preselection of the remaining images by 16 authors (AS did not contribute his assigned dataset and dropped out of the analysis from here on out), followed by a second round of image confirmation/rejection by one rater (WR), 249 images, 38 rural and 211 urban images, were retained (~ 0.23% of the total images and ~ 1.3% of the 19,495 unique contributors; for an example, see Fig. 1B).

### Snout/skull length measurements

All image measurements were done in Fiji/ImageJ (version 2.14.0/1.54f; a freeware software tool used in image analysis) and added to the data set via the corresponding image ID. Due to all the images lacking reliable scaling opportunities, snout length measurements were taken in relation to the length of the skull. The dense fur and ear tip orientation often make the end point of the skull impossible or unreliable to detect and measure. Instead, we chose the pinna attachment areas as a reliable proxy (Fig. 1). Snout length was measured from the most rostral tip of the nose to the tear duct opening. The proxy for skull length was measured from the most rostral tip of the nose to the lower and upper pinna–skull attachments. These two measurements were then averaged to represent the most reliable measurement from the nose to the center of the ear, i.e., ear canal opening. To calculate the snout-to-skull ratio, we divided the snout length by the average skull length proxy.

Prior to these measurements being taken by the authors, 13 images from the dataset were selected by the senior author to measure interrater reliability across all the raters. Interrater reliability was established through the icc function from the irr package [25]. The interrater reliability was 68% (95%-Confidence Interval for ICC Population Values: 0.509 < ICC < 0.858; p-value:1.36×10^–42^).

### Statistical analysis and data compilation

All data analysis and cleaning was completed in R (version 4.4.0) and R Studio (version 2023.09.0). The data provided via iNaturalist include coordinate, county, state, and year information, among many other identifiers. We used the county and state information provided with the images to access two data repositories: (I) the 2020 U.S. census information (https://www.census.gov/data.html) and II) the U.S. Department of Agriculture 2023 USDA Plant Hardiness Zone Map (https://planthardiness.ars.usda.gov/).

We used the census information to extract data on the rural–urban continuum code, a measure of population density and closeness to metropolitan areas divided into nine levels: levels one through three were combined into the “urban” category, and levels four through nine were combined into the “rural” category; this categorization is commonly used via the U.S. Department of Agriculture (https://www.ers.usda.gov/data-products/rural-urban-continuum-codes/documentation/). The rural–urban continuum code was first established in 1975 and has since been updated to reflect the current version used in this present study [20]. Level one describes counties with a population size of one million or more, level two describes counties with a population size between a quarter of a million and a million, and level three a county of a population size of less than a quarter million. These three categories are considered metropolitan counties by the USDA and referred to as “urban” in our study. The remaining levels were combined into this study’s “rural” category, which the USDA collectively refers to as nonmetro areas. Level four describes a county with a population of 20 000 that is adjacent to a metropolitan area. In contrast, level nine describes a county with a population of less than 5 000 that is not adjacent to a metropolitan area.

To capture the climate across the contiguous United States we used the updated 2023 USDA Plant Hardiness Zone Map. This map that was originally designed to categorize climates in the United States and divide them into zones with similar conditions [10]. The map is divided into 13 climate zones across the entirety of the United States territory based on the “average annual extreme minimum winter temperature”, which is provided in ranges of 10 degree Fahrenheit increments. Zones 4 to 11 are present in our dataset. We want to highlight that we are aware of potential issues surrounding the use of a map derived from a non-SI units. We actively chose to use this method of climate categorization over Koeppen–Geiger because it provides a more nuanced view of the climate, especially in the East, where most of our data is located [26]. We used county and state information associated with every image to extract the corresponding climate value. Lower numbers represent colder climates and higher numbers represent warmer climates.

With all data extracted and compiled we then used linear models (lm from the stats package) to create both full and null models for the complete data set and a data set of only the recent years ranging from 2020 to 2024 (N = 172). The null model for all models included only the average of the data distribution. All full models were tested against their null models and only further analyzed if they were a significant improvement and passed the following model assumptions: low variance inflating factors, stability, homogeneity, and normality of residuals. The full model for the complete data set included the dependent variable snout–skull ratio and the predictor latitude, USDA climate category, and year nested via the rural/urban category. While this model was a significant improvement over the null model and passed most model assumptions, it indicated a high variance inflation (VIF) due to the factor year. Due to the low number of rural data points in earlier years we chose to run our reduced model on the most recent data (2020–2024) only, allowing us to remove the factor year from our model and address the high VIF. This new model included the predictors latitude, USDA climate zones, and the rural/urban category. This model tested significantly better than the null model and fulfilled all assumptions. All code and data is publicly available (see: Availability of data and materials link osf.io/56xcg).

## Results

**Fig. 2:**
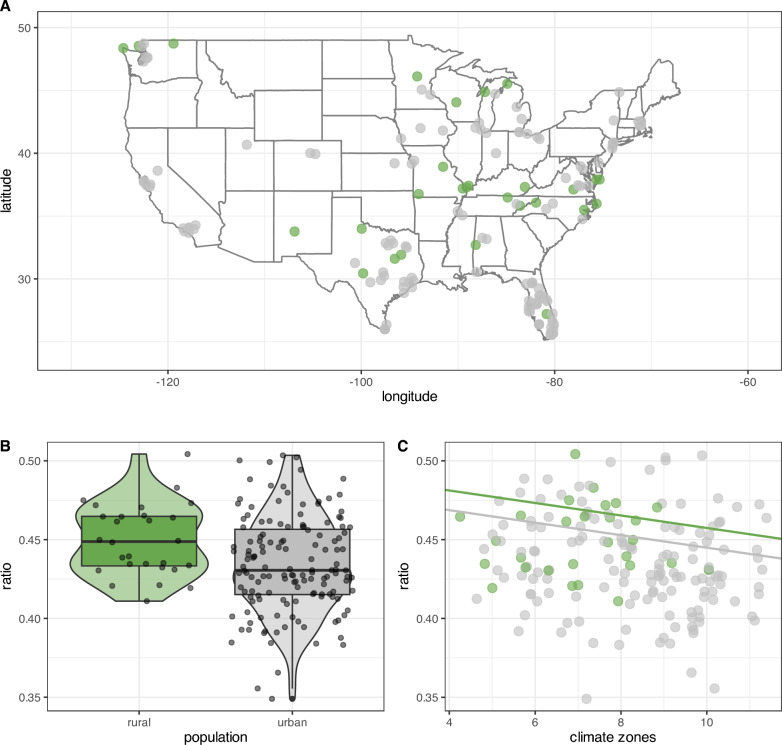
Green dots represent data from raccoon images collected in rural environments, and gray dots represent data from images taken in urban environments. Smaller ratios are indicative of shorter snouts. The data in the plots is limited to the most recent years from 2020 to 2024. **A** Visualization of all data points contributing to the analysis as located on the contiguous US map. **B** Boxplot of the snout-skull ratios of urban and rural racoons across all data points. **C** Data distributions of both rural and urban raccoon snout–skull ratios over US contiguous USDA climate zones. The solid lines illustrate the model estimates. Climate zones are displayed on the x-axis with average temperatures increase with increasing numbers. To improve readability points of each climate zone are jittered along their corresponding x-axis position

In this study, we examined whether continued environmental exposure to urban environments triggers traits consistent with the domestication syndrome. Specifically, we tested whether (continued) close proximity to human environments would result in phenotypic changes to the snout length of raccoons. Both our complete model, which included the variable year, and our reduced model were significant improvements over the null model. The high variance inflation factor (≥ 10) of the complete model (Table 1) requires caution when interpreting the main effects. Therefore we refrained from interpreting this model’s data. The reduced model without the variable year (Table 2) showed that the rural urban continuum and the USDA climate zones were significant predictors of snout length, but geographical latitude was not. We found a clear reduction in snout length in urban raccoons compared to rural raccoons (Fig. 2B, Table 2) across the contiguous United States (Fig. 2A). Controlling for climate zones, we found that both rural and urban snouts decrease in length with warmer climates (Fig. 2C). Yet, across climate zones urban raccoons have shorter snouts than their corresponding rural counterparts (Fig. 2B). Overall, we observed a 3.56% snout reduction between rural to urban raccoons.

**Table 1:** Model summary and null/full model comparison of the model comprising the influence of the urban/rural environment, USDA climate zone, latitude, and year (nested by rural and urban environment) on the snout–skull ratio. The model includes all the data and is not limited to recent years. This model was not interpreted further due to a high variance inflation factor

Snout-skull ratio all years				
*snout-skull ratio* ~ *urbanRural/year* + *USDA_Climate* + *latitude*				
	Pr(> F)			
full-null comparison	0.002171			
	estimate	standard error	t value	Pr( >|t|)
intercept *	−6.8488155	2.5364056	−2.7	0.00742
urban	8.8971406	2.757011	3.227	0.00142
USDA climate zone	−0.0043358	0.0016288	−2.662	0.00829
latitude	−0.0005984	0.0004143	−1.444	0.14997
rural:year	0.0036343	0.0012544	2.897	0.00411
urban:year	−0.0007696	0.0005477	−1.405	0.16127

*intercept includes rural raccoons

Residual standard error: 0.02861 on 243 degrees of freedom; Adjusted R-squared: 0.05464

F-statistic: 3.867 on 5 and 243 degrees of freedom

VIF ≥ 10

**Table 2:** Model summary and null/full model comparison of the model comprising the influence of the urban/rural environment, USDA climate zone, and latitude on the snout–skull ratio. The model only includes recent years ranging from 2020 to 2024

Snout-skull ratio recent years (2020–2024)				
*snout-skull ratio* ~ *urbanRural* + *USDA_Climate* + *latitude*				
	Pr(> F)			
full-null comparison	0.01145			
	estimate	standard error	t value	Pr( >|t|)
intercept *	0.49704	0.0315422	15.758	< 2*10^-16^
urban	−0.0124846	0.0061887	−2.017	0.0453
USDA climate zone	−0.0039664	0.0019605	−2.023	0.0446
latitude	−0.0005062	0.000507	−0.998	0.3195

^*^intercept includes rural raccoons

Residual standard error: 0.02895 on 168 degrees of freedom; Adjusted R-squared: 0.04676

F-statistic: 3.796 on 3 and 168 DF, p-value: 0.01145

## Discussion

In this paper, we set out to test whether urban environments lead to the emergence of phenotypic traits associated with the domestication syndrome. We predicted that urban raccoons would have shorter snouts than rural raccoons across the contiguous United States. Our data indicates the prevalence of early-stage domestication symptoms in urban areas and underscores the fact that domestication-related effects do not exist in a vacuum, but have to be considered alongside other environmental selection pressures, such as climate gradients.

Aside from potential developmental impacts on skull anatomy our results highlight the need to consider environmental factors and selection pressures on a population’s phenotype, even when the main focus of the study is an entirely different and specific selection pressure (e.g. in this case the adaptation to the human niche). Our results showed a pattern of shorter snouts in urban raccoons compared to rural ones, yet climate impacted snout length overall. Urban raccoons in cold climates have shorter snouts than their rural counterparts, but they may have longer snouts than rural raccoons from very hot climates. Bergmann’s rule states that larger body sizes are an adaptation to heat retention in colder climates. However, this rule has been relativized to also be influenced by species size, migration patterns, and other adaptations to cold temperatures such as plastic skull size in common shrews [6, 19, 40]. Despite discussions surrounding the applicability of this rule it nevertheless suggests that the differences in absolute snout length across the climate gradient could be connected to adaptations that improve heat retention.

Research on wild foxes and wild mice, as well as our data on raccoons presented here, reveals that urban animals have shorter snouts than their rural counterparts [13, 32]. Parsons and colleagues’ study (2020) of foxes revealed a distinct reduction in muzzle size in London foxes compared to those from surrounding areas. Geiger and colleagues’ study (2018) of a wild mouse population in Switzerland found that phenotypic changes associated with the domestication syndrome (i.e. white patches and shorter head lengths) increased in frequency over generations. These studies are crucial for understanding the emergence of the domesticated phenotype in wild populations. The mice results however are restricted to hyperlocal data, leading to a higher probability of relatedness amongst individuals, as the study was conducted at a barn in Switzerland. Here, we present new data on raccoons collected across various climate zones and locations; however our results mirror the patterns observed in these other species. We see a clear emergence of a phenotypic trait consistent with the domestication syndrome in urban environments.

Our results stand in support of the NCDS hypothesis and its predictions about changes in the anatomy and morphology of domesticated animals. The NCDS hypothesis states that one mechanistic pathway has the ability to capture, explain, and predict changes across a vast array of species across the phylogenetic tree [45, 46]. One such derived prediction is a reduction in snout length, a prediction with which our raccoon data aligns. However, the NCDS hypothesis does not claim that all traits of the domestication syndrome (such as snout length reductions) apply wholistically to all domesticated species. Foxes, mice, and raccoons exhibit reductions in snout length, yet the contrasting absence of a reduction in cats provides for an interesting opportunity to explore counteracting or exacerbating selection pressures in the context of (early-stage) domestication research [13, 27, 32].

While raccoons, foxes, and cats all fall under the order Carnivora, they are each members of distinct families: cats are members of the Felidae family, foxes are members of the Canidae family, and raccoons are members of the Procyonidae [1]. Both foxes and cats, as well as raccoons, would have found themselves on a commensal pathway to domestication, an active adaptation to living in a human-centric environment. Therefore, how could the same environment result in such distinctly different outcomes in terms of snout length if the mechanistic pathway is the same? Quite often, domestication might be oversimplified by putting it into a vacuum of existence, with the assumption that the selection pressures exerted by the process of domestication do not interact with other existing selection pressures. We speculate that cats that lack a reduction in snout length via the domestication process should be viewed from a species-specific context: cats already have a relatively short snout, resulting in increased bite force yet reduced olfactory ability compared with those of canines [41]. This counteracting selection pressure toward maintaining bite force but not further reducing the olfactory senses could explain the lack of secondary snout reduction in cats. Raccoons, in the family Procyonidae, are much closer related to the Canidae family and more similar in dentition and snout proportions, possibly allowing more flexibility to other evolutionary (and/or domestication related) selection pressures [30].

The NCDS hypothesis links all changes relevant to the process of domestication to the neural crest, a pivotal structure in vertebrate and even Deuterostomia evolution [12, 14, 36]. If mammals experience changes in the proliferation and migration of neural crest cells during domestication, it would be safe to assume that other vertebrates would experience the same or similar patterns; shared/similar developmental pathway biases (due to a common ancestor) combined with shared selection pressures for tameness should result in similar phenotypes [38]. Research on birds, specifically finches, has highlighted depigmentation and changes in behavior and physiology as a result of selection for tameness [31, 39]. Research on reptiles in urban environments indicates more plastic responses and pattern changes in comparison with mammalian results: while some research suggests that snouts, or rather head length, increases in length in urban environments compared with rural environments, other research highlights opposing changes [4, 42, 43]. Initial assumptions concerning the vastly different mechanistic functions of the neural crest in mammals compared with non-avian and avian reptiles do not seem to hold, as the neural crest is equally relevant to developmental processes in both avian and non-avian reptiles [9, 24].

Assuming that the mechanistic pathway of domestication (i.e., neural crest cell involvement) is indeed equivalent across the amniotic phylogenetic tree, the human environment, i.e., the human niche and selection for tameness, might not be equally impactful across species. Larger, more noticeable animals (such as raccoons), as well as species perceived as potential threats or pests, might experience more stringent domestication-related selection pressures. Smaller animals, if not threats/pests to human life and property, could be invisible from a human perspective, reducing the intensity of domestication-related selection pressures. Alternatively, selection pressures for tameness might equally affect all species and change the migration/proliferation of neural crest cells, yet kinetic skulls (in the case of reptiles and birds) and plastic responses to other environmental pressures might counteract these early changes in later stages of ontogeny [3, 23].

In summary, our data indicates the emergence of a snout reduction phenotype in urban areas that matches the traits of the domestication syndrome; this finding supports the NCDS hypothesis. These results are crucial for discussing and understanding the impact of domestication-related changes on not only current domesticates but also yet undomesticated species. We want to highlight raccoons as a new opportunity for observing early-stage domestication patterns in a mammalian model system with no possibility of introgression and hybridization with other already domesticated mammal species.

## Conclusion

In this paper we find that raccoons in close contact with densely populated human environments experience a reduction in snout length. Our data support the mechanistic pathway of the domestication syndrome outlined in the Neural Crest Domestication Syndrome hypothesis and illustrate the potential of the raccoon as a new model species to study the effects of early stage domestication processes. We also highlight the importance of considering that selection pressures associated with domestication events do not exist in a vacuum but interact with other environmental selection pressures.

## Data availability and materials

The dataset (and code) supporting the conclusions of this article are available in the OSF repository: Lesch, R. (2024, November 17). Tracking domestication signals across populations of North American raccoons (Procyon lotor) via citizen science-driven image repositories. Retrieved from osf.io/56xcg.
